# A Mixed-Methods Evaluation of the Afghan Evacuee Resettlement Programme in Aotearoa New Zealand

**DOI:** 10.1007/s10903-025-01752-4

**Published:** 2025-09-02

**Authors:** Nadia A. Charania, Irene Zeng, Priyanka Kumar, Claudia Gaylor, Eleanor Holroyd

**Affiliations:** Auckland University of Technology, Auckland, New Zealand

**Keywords:** Aotearoa New Zealand, Mixed-methods, Afghanistan, Evaluation, Humanitarian evacuation, Resettlement

## Abstract

In 2021, following the Taliban’s control of Afghanistan, Aotearoa New Zealand (NZ) welcomed over 1,700 Afghan nationals at risk due to their association with NZ agencies. The New Settlers Family and Community Trust (NFACT) was contracted to provide tailored resettlement support. This study evaluated NFACT’s Afghan evacuee resettlement programme using a mixed-methods approach, including a cross-sectional survey, interviews, and focus groups with Afghan evacuees and NFACT staff. Of the 101 surveyed evacuees, most (93.1%) expressed satisfaction with the support they received, and almost all (97.0%) would recommend NFACT’s programme to newcomers. Analysis across integration domains revealed consistently high ratings for services and support. Qualitative findings from Afghan evacuees (n = 12) highlighted the challenges of adjusting to new systems, the crucial guidance provided by NFACT, and the need for ongoing, tailored support to foster their dreams and sense of purpose. NFACT staff (n = 11) emphasised their dedication to support evacuees beyond contractual obligations, how limited resourcing constrained their efforts, and how their shared values and experiences underpinned the success of the programme. The findings underscore the need for comprehensive, culturally sensitive, and sustainable resettlement programmes. These findings have implications for governments to strengthen support for humanitarian evacuation responses within the evolving geopolitical landscape.

## Introduction

In 2021, many countries rapidly evacuated people from Afghanistan in response to the humanitarian crisis that unfolded when the Taliban took control of the country after international forces departed. Government-led humanitarian evacuations have been used as a last resort during extreme crises for multiple reasons, including civilian protection and international relations [42]. Humanitarian evacuations are complex, large-scale inter-agency operations, marked by political, ethical, and logistical challenges [42]. Globally, government-led humanitarian evacuations have varied substantially in the pathways and support provided, often reflecting inequities in policies, and generally remains an understudied area [20].

Beginning in August 2021, Aotearoa New Zealand (NZ) evacuated more than 1,700 Afghan nationals on critical purpose visas, offering them residency pathways and tailored assistance due to their risk of harm from working with NZ agencies [12, 32]. Afghan evacuees were not formally granted refugee status by the NZ Government and were offered different resettlement support compared to refugees entering NZ via other pathways [13]. For instance, only quota refugees entering via the UNHCR pathway are offered an orientation programme at Te Āhuru Mōwai o Aotearoa (Māngere Refugee Resettlement Centre) and resettlement support in the community. However, evacuees were included in the 2023 refresh of the national Refugee Resettlement Strategy along with other forcibly displaced groups, including family support and convention refugees, reflecting the nation’s broader commitments to resettlement [13]. As such, herein, we position Afghan evacuees as having refugee-like backgrounds given their experience of displacement that contributes to vulnerabilities that require support from the receiving country. While we note that Afghan evacuees may share similarities with other refugee-background groups, they may also differ; for example, in terms of demographic characteristics (e.g., education and language proficiency) and time spent in resettlement processing, which may have shaped their resettlement experiences and expectations.

The New Settlers Family and Community Trust (NFACT) was the only contracted charitable organisation by the Ministry of Business, Innovation and Employment (MBIE) to deliver a resettlement programme to over 1,700 Afghan evacuees. NFACT fosters belonging and integration, with a key focus on supporting individuals through services “*for refugees, by refugees*” [26]. Many of NFACT staff are former refugees and many are from the same ethnic background as the Afghan evacuees. All evacuees were supported with levels of support tailored to their needs starting in October 2021. NFACT’s contracted services included connecting evacuees with local government, health, mental health, language, and education services, fostering cultural connections, and advocating for their needs. Upon engagement, NFACT identified needs for further services, including parenting and resilience programmes, mental health support, employability courses, small business development, digital literacy training, driver’s license support, and access to cultural events and leadership opportunities, which were funded by a range of government agencies and philanthropic funders [25]. Moreover, NFACT staff recognised strong alignment in the desired resettlement outcomes to Māori (Indigenous people of NZ) holistic models of health and wellbeing, such as Te Whare Tapa Whā and Te Pae Mahutonga [16, 17]. As such, principles related to the multiple dimensions of health including spiritual (wairua), family (whānau), physical (tinana), and mental (hinengaro), were embedded within NFACT’s framework, while also fostering opportunities to strengthen relationships between Māori and Afghan evacuees.

Given the variability in humanitarian evacuation efforts, along with the need to identify those that most effectively promote positive outcomes, evaluating humanitarian evacuation programmes is essential. As such, this mixed-methods study evaluated NFACT’s Afghan evacuee resettlement programme and developed recommendations to improve resettlement programmes in the context of humanitarian crises. Our presented study builds on previous findings that focused on Afghan evacuees’ resettlement experiences to advocate for improved support for those resettling for humanitarian reason in NZ [30].

## Materials and Methods

This study used a parallel convergent mixed-methods design [6]. This study represents a community-academic partnership between NFACT and researchers from the Auckland University of Technology. An Advisory Group was specifically set up to guide the presented study. Researchers collaborated with two NFACT stakeholders and four Advisory Group members. Members were invited based on recommendations from the research team and two NFACT stakeholders based on their local knowledge, experiences, and expertise. The group included one Māori individual and three individuals from refugee backgrounds, two of whom are respected Afghan community leaders. They contributed to the development of the study instruments and reviewed preliminary findings. Advisory Group members received a koha ($50 gift voucher) in appreciation of their time and input. Ethical approval was granted by the Auckland University of Technology Ethics Committee (23/160).

### Transformative Research Paradigm

This research was guided by the transformative research paradigm which emphasises social justice and human rights in knowledge production [6, 22]. A key feature is the inclusion of diverse stakeholders throughout the research process in a respectful, reciprocal, and transparent manner [21, 22]. This suited our collaborative research and evaluation project that aimed to improve support for resettled populations, a historically marginalised group. The paradigm’s axiological assumptions recognise power imbalances and how these can contribute to societal inequities. We were attuned to how intersecting social identities related to gender, ethnicity, education, and socioeconomic status, to name a few, contributed to oppression and privilege depending on the context. Within this paradigm, while all versions of reality are valued, they are critiqued to understand the role of power in perpetuating inequities [22]. As such, knowledge is viewed as ever-changing and reflects the product of social processes and power structures [22]. We also recognised the interrelated researcher-participant relationship and potential power imbalances when generating data [22].

### Development of Study Instruments

We drew from the ten domains of integration included in the Refugee Integration Survey and Evaluation (RISE) survey [31], which operationalised the conceptual framework of Ager and Strang [1] that includes dimensions related to markers and means (employment, housing, education, health), social connection (social bridges, bonds, and links), facilitators (language and cultural knowledge, safety and stability), and foundation (rights and citizenship) to understand refugee integration. More specifically for social connection, social bonding refers to horizontal interactions with people from the same culture/ethnic group, social bridging refers to horizontal interactions with people from different cultural/ethnic group, and social links refers to vertical relationships that link individuals or groups to higher-level entities such as government agencies [31]. This framework has guided the evaluation of refugee services in other countries, including Australia [45].

For the survey, two to five research and evaluation questions were posed within each domain of integration using the item concepts from the RISE survey as a guide. To evaluate the overall programme, additional questions were added about what support they would have like offered, if they would recommend the programme to other newcomers, and overall satisfaction with the programme [27, 33]. The primary research outcome was related to social connections as this directly supports integration and can be supported through resettlement programmes. The survey was developed in English, translated into Dari, and back-translated for accuracy. The Advisory Group were consulted to provide feedback regarding clarity, relevance, and appropriateness of the wording. Members suggested adding some more prompts and improve the clarity of the study context. An interpreter was recruited through the contacts of NFACT staff and was proficient in Dari, Pashto, and English. Two members of the research team (NC and EH) provided training to the interpreters covering the study’s purpose, design, and best practices for administering the survey and conducting the interviews. One of the interpreters piloted the survey with one individual with an Afghan cultural background which was well received and as such, no further changes were made.

The evacuee participant interview guide was also based on the work of Ager and Strang [1]. Open-ended questions were used to gain insights into the type of resettlement support evacuees needed and ways in which NFACT’s programme met or not met their needs. The interview guide for NFACT staff focused on their experiences with developing and delivering the programme. The Advisory Group vetted the guides to ensure cultural appropriateness and alignment with the study's aim. Modifications included adding culturally-appropriate greetings, background context, and clarifying question wording.

All participants also completed a brief form to collect information about key sociodemographic variables. As a token of our appreciation, all participants received a koha (gift voucher).

### Quantitative Phase

#### Data Collection

The cross-sectional survey assessed the resettlement needs and experiences within a cohort of 1,741 Afghan evacuees, 78% of whom were resettled in the geographical region of Auckland [30]. Participants were eligible if they were over 18, entered NZ via the Afghan evacuee pathway, lived in Auckland for 3–6 months, and were proficient in English, Dari, or Pashto. Those related to NFACT staff were excluded. Recruitment was mainly through NFACT via flyers, newsletters, social media, and verbal invitations.

Participants who were interested contacted the designated interpreter. After confirming their eligibility and obtaining informed consent, the interpreter conducted the survey via telephone or in-person in their preferred language. Survey responses were entered into RedCap directly by the interpreters. We recruited a total of 102 adult participants between November and December 2023. One participant was ineligible as they were related to an NFACT staff member, leaving 101 participants for analysis.

#### Data Analysis

Demographics were analysed using descriptive statistics. As we wanted to test the association between demographic factors and social connectedness, we used the Cochran-Armitage Trend Test, and also included the other covariates using generalised logistic regression. Evaluation questions were also summarised, with demographic differences tested using the same methods. The expected participation rate in aspects of NFACT’s programme were compared against a benchmark suggested by the Advisory Group (75%). Due to the discrepancy in gender distribution of the sample compared to the total Afghan evacuee population in NZ, post-stratification adjusted for sampling and non-response bias based on the gender distribution was conducted [30]. Analysis was conducted using SAS, R, and SPSS. Responses to the open-ended questions were manually analysed using a qualitative summative content analytic approach [11].

### Qualitative Phases

The two qualitative phases were guided by a qualitative descriptive methodology [37, 38] and involved semi-structured interviews with Afghan evacuees and focus groups with NFACT staff.

#### Researcher Positionality

Recognising the importance of reflexivity [3], we (NC and PK) engaged in informal discussions with each other and the team to reflect on our positionality throughout the study [8]. With a background in public health and over 15 years of qualitative research experience, NC, an ethnic-minority migrant, was particularly attuned to evacuees’ conceptualisations on meaningful opportunities from a religious and cultural perspective. PK’s experience in public health research and personal migration background enabled insights into the complexities of integration, including the challenges with navigating new cultural norms, identify shifts, and balancing traditional and host country values.

#### Data Generation

In November 2023, 12 Afghan evacuees participated in in-person semi-structured interviews. Survey participants were invited for follow-up interviews, and were purposefully selected to help ensure diversity based on age, gender, education, and language proficiency. All interviews were conducted in English, with a professional interpreter providing consecutive language interpretation. The interviews took place at NFACT’s office, a familiar and convenient location for participants, and lasted 30–60 min.

In August 2023, 11 NFACT staff members participated in focus groups or interviews. Eligible staff were over 18, proficient in English, employed for at least six months, and involved in the Afghan evacuee programme. Line managers were excluded to ensure a safe space to share experiences. In-person sessions were held at NFACT’s office while interviews were done online. Focus groups lasted about 90 min, and interviews lasted an hour.

Based on literature and prior work, we determined the final participant number was sufficient to address the study’s aim and feasible within logistical constraints [3, 36].

#### Data Analysis

Interviews and focus groups were audio recorded with participants' consent and transcribed verbatim. NC and PK checked the transcripts for accuracy. NC analysed the Afghan evacuee data, while PK analysed the NFACT staff data. Manual coding and using software (e.g., NVivo) were used to support data management and analysis.

Data were analysed following the five-step conventional content analytic approach. First, we familiarised ourselves with the data by listening to the audio recordings and reading the transcripts. Then, we inductively coded the data word-by-word, creating an initial coding scheme. From this, codes were organised to create categories and sub-categories. Finally, we refined and defined each category, sub-category, and associated codes, and included example quotes [11].

#### Rigour

Assessing the rigour of this mixed-methods research was guided by the rigorous mixed methods framework. We outlined the parallel convergent design, its value, and reported data collection and analysis procedures for both strands [10]. For each strand, we also attended to methodologically specific elements of rigour. The developed survey undertook a process for face and content validity (i.e., clarity, culturally appropriateness, internal consistency) [19]. The qualitative phase followed the four criteria of trustworthiness (credibility, conformability, dependability, and transferability) [15]. We provided a "thick description"of research decisions and engaged in reflexivity, particularly during analysis. Preliminary findings were discussed with the research team as a form of peer debriefing.

## Results

### Findings from the Quantitative Survey with Afghan Evacuees (N = 101)

#### Description of the Sample

##### Demographic Characteristics

Of the 101 sampled evacuees, most were female (65.3%), younger adults (72.3% were younger than thirty-nine years old), and of Hazara ethnicity (56.4%) (Table 1). Female participants were over-represented compared to the total female population of evacuees that arrived in NZ (49%). Female respondents were slightly younger than their male counterparts. More than half of the surveyed evacuees had three or more children (57.4%), while 28.7% had no children. Evacuees predominantly settled in Auckland CBD (59.4%), followed by Auckland’s other districts (41.6%).

**Table 1:** Demographics, education, employment, training, housing status, and general health of participating Afghan evacuees (N = 101)

Demographics	N	Percent	Education, employment, and housing	N	Percent	Subjective health by age	Mean	N	SD
Age			Highest level of education			18–29 years	4.7	31	0.7
18–29 years	31	30.7%	School qualification	15	14.9%	30–39 years	3.7	41	1.1
30–39 years	42	41.6%	Bachelor’s or postgraduate degree	38	37.6%	> 39 years	3.6	28	1.2
> 39 years	28	27.7%	No qualification	21	20.8%	Total	4.0	100^ƚ^	1.1
Gender			Other	27	26.7%	(1 = very poor, 5 = very good)			
Female	66	65.3%	Current education in Aotearoa New Zealand						
Male	35	34.7%	Currently studying or completed	45	44.6%				
Ethnicity			Not studying	56	55.4%				
Hazara	57	56.4%	Done paid work in a job or business in Aotearoa New Zealand^ƚ^						
Tajiks	19	18.8%	Yes	15	14.8%				
Other^*^	25	24.8%	No	85	84.2%				
Number of children			Current employment status^**ƚ**^						
No children	29	28.7%	Full-time (> = 30 h)	5	5.0%				
1–2	14	13.9%	Part-time (< 30 h)	2	2.0%				
3	28	27.7%	Not in paid employment	93	93.0%				
> 3	30	29.7%	Satisfaction with current home						
Settlement location			Satisfied or very satisfied	68	67.3%				
Auckland CBD	60	59.4%	Neutral/unsure	13	12.9%				
Other Auckland Districts	41	41.6%	Somewhat or very dissatisfied	20	19.8%				

SD = Standard deviation

* Sadat, Uzbek, and Pashtun ƚ Prefer not to answer or missing value n = 1

##### Employment, Education, and Training

A substantial proportion of respondents held a bachelor’s or postgraduate (Master’s, Doctorate) degree (37.6%) (Table 1). Almost half (44.6%) were attending or had completed studies or job training since arriving in NZ. In contrast, only a minority had a paid job at the time of data collection (7.0%) or had ever done paid work in a job or business since arriving in NZ (14.8%). Most study participants reported having difficulties finding a job, both now and in the past (89.0%).

##### Health

Surveyed evacuees reported being in good health (mean = 3.97, SD 1.11) on a 5-point scale (1 = very poor, 5 = very good) (Table 1). Younger respondents aged 18 to 29 reported better general health than those aged 30 years and older (mean = 4.7 versus 3.7 and 3.6).

##### Housing

Over half of the surveyed evacuees were satisfied with their current homes in relation to aspects, such as the number of rooms and proximity to shops, schools, childcare, and public transport (67.3%) (Table 1). However, when asked what additional support evacuees would have liked NFACT to have offered, support in finding appropriate housing and information on tenancy rights and responsibilities were among the most common needs mentioned by study participants.

##### Language, Cultural Knowledge, and Social Connections

Overall, the findings suggest that evacuees considered themselves to have good English language proficiency and reported a strong sense of belonging to the community in NZ (mean = 4.4, SD 0.96) on a 5-point scale (1 = never, 5 = always) (Table 2). Evacuees with school or higher qualifications demonstrated a higher level of English language proficiency than those with less education (mean = 3.0, 2.9 versus 1.4). The majority were most comfortable speaking Dari, followed by English (Dari was also the most used survey language). However, understanding the local ways and culture of their new homeland continued to be challenging for 66.4% of study participants. Responses also indicated that it was easier to establish social connections with people of the same culture and/or ethnic group than to build friendships across cultures (57.4% versus 24.7% rating it easy or very easy). It is worth noting that many evacuees found it difficult to make friends in general, whether within the same or across different cultural and/or ethnic groups.

**Table 2:** Language, cultural knowledge, and social connection among participating Afghan evacuees (N = 101)

	N	Percent/Mean	SD	Min	Max	Median (IQR)
Language						
English language proficiency (1 = not at all, 4 = very well)	101	2.5	0.91	1	4	3 (2–3)
School qualification	15	3.0	0.38	2	4	3 (3–3)
Bachelor's or postgraduate	38	2.9	0.69	1	4	3 (2.75–3)
No qualification	21	1.4	0.67	1	3	1 (1–2)
Other	27	2.4	0.84	1	4	3 (2–3)
Language comfortable speaking (select all that apply)						
Dari	96	60.0%				
English	33	20.6%				
Pashto	26	16.3%				
Other	5	3.1%				
Social connection						
Feel part of the community in NZ (1 = never, 5 = always)	101	4.4	0.96	1	5	5 (4–5)
Understanding of local ways/culture						
Easy or very Easy	28	27.7%				
Neutral/unsure	6	5.9%				
Hard or very hard	67	66.4%				
Making friends with people from the *same* culture/ethnic group (social bonding)						
Easy or very Easy	58	57.4%				
Neutral/unsure	3	3.0%				
Hard or very hard	40	39.6%				
Making friends with people from *different* culture/ethnic group (social bridging)						
Easy or very Easy	25	24.7%				
Neutral/unsure	5	5.0%				
Hard or very hard	71	70.3%				

SD = Standard deviation. Min = Minimum. Max = Maximum. IQR = Interquartile Range

##### Rights and Citizenship

All evacuees who participated in the study intended to apply for citizenship in NZ.

#### Demographic Factors and Social Connectedness

The survey included four questions that assessed social connectedness and were analysed using generalised logistic regression. Both English-speaking proficiency and age were significantly associated with participants’ likelihood of making friends with individuals from different ethnic groups (p-value: 0.0094 and 0.025, respectively), as well as their ease of understanding the local culture (p-value: 0.0045 for English proficiency). No differences based on gender were found. Participants who reported speaking English well and very well had less difficulty making friends with individuals from different cultural or ethnic groups (18 (30.5%) vs 7 (16.6%)) and found it easier to understand local culture (20 (33.9%) vs 8 (19.0%)) (Table 3). Moreover, participants in the younger group found it easier to make friends from different ethnicities than those in the older groups (aOR 30–39 years vs 40 years and above: 3.6 (95% CI: 1.4–9.2). More than half of the participants found it easy to make friends within their ethnic group and felt a sense of community either always or most of the time; these experiences did not significantly vary across different age groups (p-values: 0.85 for making friends and 0.18 for sense of community) or gender (p-value: 0.25 and 0.77, respectively) and were not associated with spoken English proficiency (p-value: 0.15 and −0.66, respectively) (Table 3).

**Table 3:** English language proficiency and social connectedness among participating Afghan evacuees (N = 101)

Since you came to Aotearoa New Zealand, how easy have you found it to make friends with people from *different* cultures/ethnic groups?					Since you came to Aotearoa New Zealand, how easy have you found it to make friends with people from the *same* culture/ethnic groups?				
	English language proficiency					English language proficiency			
	Not at all or not well	%	Well or very well	%		Not at all or not well	%	Well or very well	%
Very hard/hard	34	81.0%	37	62.7%	Very hard/hard	19	35.3%	21	35.6
Neutral/Unsure	1	2.4%	4	6.8%	Neutral/Unsure	1	2.4%	2	3.4%
Easy/very easy	7	16.6%	18	30.5%	Easy/very easy	22	52.3%	36	61.0%
* Cochran-Armitage Trend Test p = 0.009					* Cochran-Armitage Trend Test p = 0.15				

Since you came to Aotearoa New Zealand, how easy have you found it to understand the local ways and culture?					Do you feel part of the community in Aotearoa New Zealand?				
	English language proficiency					English language proficiency			
	Not at all or not well	%	Well or very well	%		Not at all or not well	%	Well or very well	%
Very hard/hard	34	81.0%	33	55.9%	Never/hardly ever	0	0%	2	3.39%
Neutral/Unsure	0	0%	6	10.2%	Some of the time	10	23.81%	12	20.34%
Easy/very easy	8	19.0%	20	33.9%	Most of the time/always	32	76.2%	45	76.3%
* Cochran-Armitage Trend Test p = 0.007					* Cochran-Armitage Trend Test p = 0.66				

##### Post-stratification by Gender

The total cohort included 1,741 Afghan evacuees, 49% were female and 51% were male [30]. Within our survey sample, 65% were female and 35% were male. Post-stratification was conducted by deriving a gender calibrated weight. Results from weighted employment, housing, and social connectedness questions were compared to those from the unweighted questions. The difference between post-stratified results and unweighted results were less than 2% except for “understand local ways/culture”; the weighted results had less selections in “very hard” (weighted 40.2% vs. unweighted 44.6%) but more in “hard” (weighted 25.7% vs. unweighted 21.8%).

#### Programme Evaluation

Regarding the helpfulness of the support and services provided by NFACT, the analysis found high average ratings across all domains, with mean ratings ranging from 4.5 to 4.9, on a 5-point Likert scale (1 = not helpful, 5 = extremely helpful) (Table 4). All the participation rates were higher than the expected 75% benchmark, except for programmes for ongoing learning, employment, rode code (driving), and understanding rights and responsibilities. The particularly low participation rate in the rode code (driving) programme may be due to the ability to only offer limited spots for practical training and lessons.

**Table 4:** Evaluation of NFACT’s resettlement programme

Support service or programme	Number		Rating			
	N	Percent	Mean	Min	Max	Median (IQR)
*Employment, education, and training*						
Enrol child(ren) in school	84*	83.2%	4.9	4	5	5 (5 – 5)
Enrol in English classes	80*	79.2%	4.8	3	5	5 (5 – 5)
Ongoing learning (e.g. sewing, parenting or family resilience programmes, etc.)	75	74.3%	4.7	3	5	5 (4 – 5)
Programmes to support employment (e.g. digital literacy, business starter course, etc	43*	42.6%	4.5	3	5	5 (4 – 5)
*Health*						
Enrol with a general practitioner	96	95.0%	4.8	3	5	5 (5 – 5)
Health and wellbeing programmes (e.g. migrant family resilience programme, counselling, etc.)	86	85.1%	4.6	2	5	5 (4 – 5)
*Housing*						
Support in relation to housing needs	82	81.2%	4.7	2	5	5 (5 – 5)
*Social connections, language and cultural knowledge*						
Programmes and events to make friends and be more familiar with the community (e.g. cultural festivals)	83	82.2%	4.6	2	5	5 (4 – 5)
*Facilitators*						
Orientation programme	90	89.1%				
Health	85	94.4%				
Education	82	91.1%				
Work and Income	78	86.7%				
Housing/Tenancy obligations and rights	70	77.8%				
Police	68	75.6%				
Road Code (driving) programme	47	46.5%	4.9	3	5	5 (5 – 5)
*Rights and citizenship*						
Understand rights and responsibilities	69	68.3%	4.8	2	5	5 (5 – 5)

SD = Standard deviation. Min = Minimum. Max = Maximum. IQR = Interquartile Range. Likert scale 1 = not helpful, 2 = slightly helpful, 3 = moderately helpful, 4 = very helpful, 5 = extremely helpful

*Missing n = 1 (1.0%) as not applicable/support not needed/unsure

##### Health Support

In terms of NFACT services, most evacuees surveyed received support to enrol with a general practitioner (95.0%), and 81.5% received assistance through health and wellbeing programmes (e.g., migrant family resilience programme, counselling).

##### Employment, Education, and Training

Regarding support for employment, education, and training, less than half of the respondents reported receiving assistance related to their employment (42.6%) or to enhance their driving skills (46.5%). A large proportion of participants had enrolled in English classes (79.2%), and 83.2% received support to enrol their children in school.

##### Facilitators

Most participants (89.1%) took part in NFACT’s orientation programme. Among those who attended, the health module was considered the most helpful (94.4%). Modules on police and housing, tenancy obligations, and rights were also perceived as beneficial by many respondents (75.6% and 77.8%, respectively).

##### Language and Cultural Knowledge, Social Connections, Rights and Citizenship

Most participants (82.2%) attended NFACT’s programmes and events to make friends and become more familiar with the community (e.g., cultural festivals). Over half (68.3%) reported receiving support to understand their citizenship rights and responsibilities.

##### Programme Evaluation Results by Demographic Factors

Limited English language ability was significantly associated with positive evaluation of support to access health services. Participants who self-reported not speaking or speaking English poorly were more likely to rate the helpfulness of support in accessing GP services highly, with 85% rating it as extremely helpful, compared to 76% of those who self-reported speaking English well. Age was also significantly associated with evaluation of English education support; participants aged 40 and over were more likely to rate this support highly compared to younger participants. The other evaluation questions were not significantly associated with any of the collected demographic variables.

#### Overall Satisfaction with NFACT’s Resettlement Programme

**Fig. 1:**
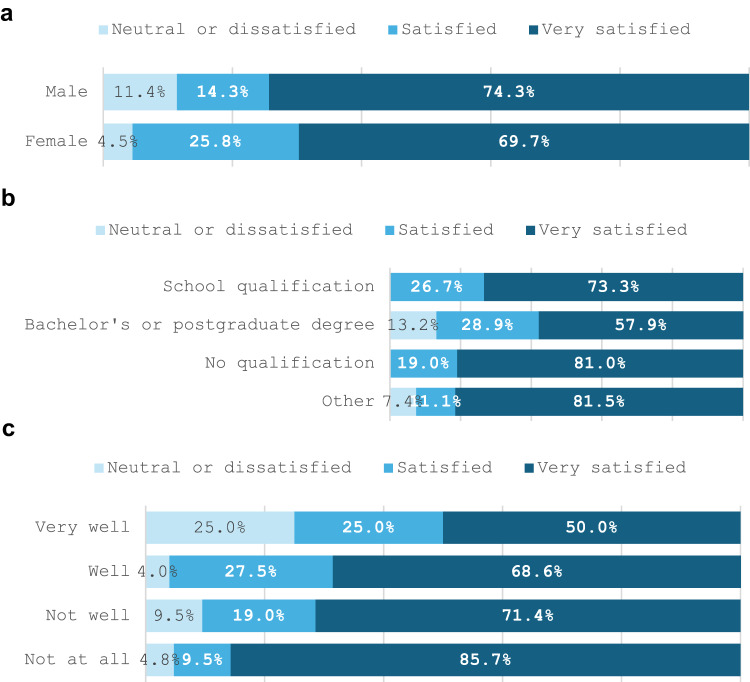
**a** Overall programme satisfaction rating by gender. **b** Overall programme satisfaction rating by educational qualification. **c** Overall programme satisfaction rating by English language proficiency

Overall, 97% of respondents would recommend NFACT's settlement support services to newly arrived refugees and those with refugee-like backgrounds in NZ. Most respondents were satisfied or very satisfied with the settlement support they had received from NFACT (93.1%). Overall satisfaction by demographic characteristics show slight differences by gender, education, and language proficiency (Fig. 1a–c).

Further support that was seen to be needed from NFACT included assistance in finding suitable housing and information on tenancy rights and responsibilities. Responses also indicated that improvements were necessary in programmes delivering employment and skills training (e.g., driving courses, assistance with job searching). However, the feedback did not specify the exact nature or scope of the recommended improvements. Additionally, just under a quarter of evacuees emphasised the importance of continuous and long-term settlement support. Some respondents also emphasised the need for support in financial matters, political education (e.g., learning about human rights and democracy), and digital literacy.

### Findings from the Qualitative Interviews with Afghan Evacuees (N = 12)

#### Participant Demographics

The evacuees were diverse in age, gender, and ethnicity (Table 5), with many speaking Dari and two speaking English. Nearly half (41.7%) held a Bachelor’s or postgraduate degree, while others had school or trade qualifications or were studying. Despite strong educational backgrounds, most (n = 11, 91.7%) were not employed at the time of the interviews.

**Table 5:** Demographic characteristics of interviewed Afghan evacuees (N = 12)

Characteristic	Number (percent)
Age (years)	
18–29	4 (33.3%)
30–39	3 (25.0%)
40–49	3 (25.0%)
50–59	2 (16.7%)
60 +	–
Gender	
Female	7 (58.3%)
Male	5 (41.7%)
Ethnic group	
Pashtun	4 (33.3%)
Tajiks	3 (25.0%)
Hazara	4 (33.3%)
Uzbek	–
Other	1 (8.3%)
Language(s) spoken^*^	
Dari	10 (55.6%)
Pashto	5 (27.8%)
English	2 (11.1%)
Other	1 (5.6%)
Educational qualification	
School qualification	3 (25.0%)
Bachelor’s or postgraduate degree	5 (41.7%)
Trade qualification	1 (8.3%)
No qualification	1 (8.3%)
Other	2 (16.7%)
Employment status	
Full-time (30 h or more)	–
Part-time (less than 30 h)	1 (8.3%)
Not in paid employment	11 (91.7%)
Number of children	
None	3 (25.0%)
One	–
Two	1 (8.3%)
Three	4 (33.3%)
Four and above	4 (33.3%)
Annual household income (NZD)	
Less than $25,000	6 (50.0%)
$26,000-$50,000	–
$51,000-$75,000	–
$76,000-$100,000	–
More than $100,000	–
Prefer not to answer	6 (50.0%)

*Participants could select multiple options

#### Challenging Standstill

Evacuees expressed a deep sense of loss since arriving in NZ. While some evacuees stated that they were happy, this sentiment seemed to stem from their gratitude for being safe and for their families having the opportunity to build new lives in a beautiful country like NZ. Their narratives, on the other hand, reflected feelings of being lost and helpless, as they desperately missed the vibrant socio-cultural and economic aspects of their lives back home. Particularly concerning was that some evacuees had been separated from their loved ones, including their children and immediate family members, causing immense stress due to ongoing concerns for their safety.

All participants spoke about how challenging it was when they first moved into the NZ community and the pressures they felt. They were unfamiliar with life in NZ and its systems, which hindered their ability to meet their basic immediate needs. Many participants discussed how the stress and pressures of resettling affected their mental health, especially during the initial period. One participant used the metaphor of a tree being replanted to symbolise his experience and the time it takes to adjust to life in a new country:

“I felt that I'm like a tree, to take out from the root and plant somewhere else. Up to that time it become again rooted and become green, it takes a lot of time. So, when I came here, I felt that I lost everything … how can I manage all these problems?” (Interviewee #7)

A few participants mentioned that their current housing arrangements met their needs, but the majority rented houses that were described as old, cold, too small to accommodate their families, and far from schools and amenities. Participants also expressed financial concerns related to high housing rental costs. Additionally, language barriers and cultural differences posed challenges for evacuees of all ages when trying to establish social connections to support their resettlement.

“Nobody talk with us because everywhere everybody's busy. When they are talking with us, just talk two words. That's all. But because of this, we are not learning English...” (Interviewee #1)

Education was a top resettlement priority among evacuees. All the participants spoke of wanting to improve their English language skills and pursue meaningful careers in NZ. For younger participants, it was evident that the language barrier impacted their adjustment to the secondary school system and their ability to build friendships across cultural groups. Young adults and parents of young children discussed the impact of being placed in mainstream versus ESOL classes for learning English.

#### Guiding the Way

Evacuees praised the resettlement services offered by NFACT and expressed much gratitude. They appreciated support for immediate needs and opportunities to participate in various programmes, such as cultural celebrations, courses, and information sessions. Tailored programmes addressed age- and gender-specific needs were also well received, including weekly football practices for youth and sewing and quilting programmes for women. Participants’ narratives reflected how these programmes fostered belonging and social connections, while building their capabilities and confidence.

“They [NFACT] really help us because we went for camping. We met a lot of people, Afghan people and also Kiwi and European people. We learn a lot from them and also, we show to them our culture, but mixing everything, it was great.” (Interviewee #8)

It was evident that NFACT staff focused on building trusting relationships and went above and beyond to support the evacuees. Many NFACT staff members spoke Dari and Pashto and were either of Afghan descent or familiar with Afghan culture, thereby minimising cultural and linguistic barriers. Evacuees described the staff as kind and helpful, praising the warm, welcoming environment they created. Importantly, it was beneficial for evacuees to have people they could contact for support as they settled into the community. They shared examples of attempting to contact government agencies for assistance but not receiving a response or struggling to communicate their needs due to language barriers. As a well-known and credible organisation, NFACT staff acted as much-needed and culturally safe intermediary who could effectively communicate with agencies to access available support and advocate for the evacuees. Participants shared that,

“… when we know about the NFACT, we know there's [a] human being helping us. Someone there's helping us. Otherwise, nobody help us.” (Interviewee #2)

#### Support to Cultivate Dreams and Sense of Purpose

Evacuees expressed the need for continued support that would evolve over the course of their settlement journey, tailored to their individual and family needs and goals. They noted that certain aspects of resettlement, such as English language proficiency, meaningful employment, safe and appropriate housing, and the establishment of social connections, take time and require ongoing support. Many evacuees’ narratives reflected a strong desire to learn and build their capabilities and capacities, enabling them to address their resettlement needs independently and become more self-reliant.

“But I really want to learn the language, because I want to solve my problem by myself.” (Interviewee #9)

Many evacuees spoke of a loss of identity and meaning tied to their previous well-respected and influential occupations back home. Participants expressed a strong desire to find permanent employment to achieve financial stability and independence. Some suggested additional support to find employment opportunities that matched their qualifications and areas of expertise, although they were also eager to re-train in new and different fields as needed. Over time, evacuees felt this would help them assist others and contribute to NZ society, reinforcing values of autonomy, reciprocity, and collectivism. As one participant stated,

“All of us learning English. That's good. We improve our language. But in future, if we find that we have to work, because all the time, New Zealand government cannot support us. And also it's not good. We have to do something. We have to be on our own, do something by our own. We enjoy to do something, we enjoy to find the work, we really want that.” (Interviewee #6)

Overall, evacuees would overwhelmingly recommend NFACT’s settlement programme newcomers. One participant said that,

“Because when we were there, we don't know anything. We cannot speak English. The life was too hard for us. If someone like NFACT did not help us, we don't know which is the right and which is the wrong way. They help us, they support us. We are here and now we are better. But they [newly arrived individuals] also need the support like that because they don't know about the rules of New Zealand, anything. Everything, it'll be new for them, like us, it'll be too hard for them. But when they have them to show this is the way you can go, then they settle.” (Interviewee #12)

Given the positive reception of NFACT’s programmes, several evacuees suggested offering more support to further their social learning and development. Driven by a strong desire to improve, participants expressed interest in learning more about parenting skills, NZ history, local Māori culture and other cultures, language, and women’s rights. Youth expressed a desire for more opportunities to engage in team sports and learn about different cultures. Evacuees also suggested offering specific programmes for young children. Since it was culturally acceptable for women to attend programmes at NFACT, participants suggested additional programmes for women, particularly those who were illiterate or did not have access to formal education, to socialise and acquire professional skills of interest, such as sewing and beauty courses. As some evacuees missed attending programmes due to lack of awareness or insufficient spaces, they suggested better advertising and additional funding to accommodate more people. Immigration support was also requested, specifically to obtain citizenship and apply for visas for their family members still in Afghanistan.

### Findings from Focus Groups and Interviews with NFACT Staff (N = 11)

#### Supporting Evacuees to Overcome Challenges

NFACT played a vital role in ensuring that evacuees had access to essential services and felt supported. However, they discussed instances where they experienced challenges in accessing support for evacuees. For example, staff struggled to enrol some evacuees in school because they had not been granted refugee status or given relevant documentation. Moreover, due to the high needs of families, NFACT staff went beyond their contracted responsibilities. To enhance efficiency, they began offering skill-building workshops to better support multiple families.

NFACT’s proactive approach also facilitated integration by equipping families with knowledge of essential services, enabling them to navigate their interactions with the health, education, and social systems in NZ. This knowledge provided families with the opportunity to make informed decisions about their lives and actively participate in their new community.

“NFACT did something that nobody did in New Zealand and no agency did it. We are providing orientation information sessions rights and access to policing, education, Work and Income, Study Link, health sector just to inform them and know their rights as a permanent residents and how to receive the services.” (Session 1)

This level of support was crucial for building trust and confidence among evacuees, helping them feel more at home and reducing the anxiety and uncertainty often associated with resettlement. By being well-informed, families could better advocate for themselves, seek appropriate help when needed, and utilise the resources available to them to their fullest. However, as NFACT was the first point of contact for families requiring support, challenges arose for the organisation.

#### Grappling with Resourcing

As NFACT was the primary point of contact for these families, most would reach out to NFACT whenever they required support, some of which NFACT was unable to deliver on. Setting boundaries became a challenge, as families did not know where else to seek help, expressed their frustration with NFACT, and felt neglected if their needs were not immediately addressed. However, some of the requested support fell outside the organisation’s contracted requirements and working hours. NFACT staff grappled with providing support in a “one size fits all” manner as per their tasked responsibilities. Being under-resourced and acting as the frontline service for the myriad of support that evacuee families needed posed challenges of its own for NFACT staff.

“The team started becoming heavily involved with work and income issues. Heavily involved with housing issues, heavily involved with communicating with... There was lots of need for communication with the other agencies like the agency to connect our family. It didn't stop with only three [contracted] tasks.” (Session 1)

Furthermore, although the challenges that evacuee families faced were familiar to NFACT, supporting additional tasks outside of their contract required even more from their already overburdened staff. To work more efficiently within resource constraints, NFACT pivoted to provide interventions that targeted multiple families at once, such as hosting workshops and information seminars. This approach enabled NFACT not only to work effectively within their limited means but also created a space for knowledge and information sharing that drew on their dedication, determination, and resourcefulness.

The duration of funding was also a significant challenge for NFACT. As families continued their resettlement journey in NZ, their needs changed over time. However, NFACT was only contracted to support evacuee families for a year from their entry into the country.

“You know, 12 months’ worth of funding isn't nearly sufficient to help these people actually become self-reliant.” (Session 4).

Consequently, staff emphasised the importance of increased, sustainable funding to not only support families for longer but also to employ additional staff to better address the evolving needs of the evacuees. It should be noted that while NFACT faced its own set of challenges in providing support to evacuees, there were also triumphs and future plans developed.

#### Successes and Future Directions

The critical role of NFACT’s culturally and linguistically relatable staff, along with their empathetic approach, has played an enormous part in the success of the resettlement services provided. A primary marker of success was that staff shared similar backgrounds with the evacuees, allowing them to understand the evacuees’ struggles themselves and related to their current experiences. For the evacuees, this created a sense of relatability that was instrumental in fostering a caring and supportive environment for the resettled families.

NFACT’s emphasis on providing holistic and tailored support, covering various aspects of resettlement, was identified as a key factor in the overall success of the programme. NFACT’s determination to provide comprehensive support for families exemplifies their genuine care and compassion as they refrained from adopting a ‘tick-box’ approach to their contracted responsibilities, even while facing their own resourcing challenges.

“NFACT is an organisation that basically understands, feels and delivers … understand them and then deliver what they really need as opposed to somebody who would take it as a simply project delivery. Tick, tick, tick, done, done, done, complete project, that's it.” (Session 2)

Looking ahead, NFACT staff spoke of plans to secure additional funding to extend their support and offer a broader range of services. Additionally, NFACT intends to engage in advocacy for national policy changes and shifts within the resettlement space to better support the holistic integration of migrant and refugee populations. NFACT’s overarching goal is to continue enhancing the holistic support provided to resettling populations by leveraging the unique strengths of their staff, who have lived experience, through an empathetic, wrap-around approach.

“So at least to be available for families when there are challenges, but definitely you'll need ongoing support to be available to provide service, for the timeframe there needs to be kind of well established, well-structured research that for a refugee or any settlement process, a five year is very okay… the five-year is more crucial period.” (Session 1)

The findings demonstrate the importance of leveraging the unique perspectives and lived experiences of migrants and refugees in the design and implementation of resettlement programmes, as this can significantly enhance the effectiveness and impact of such initiatives.

## Discussion

Data collected from the quantitative and the qualitative phases demonstrated similarities regarding the key resettlement challenges faced by evacuees and their experiences with accessing support from NFACT. Key resettlement priorities related to health and wellbeing, housing, English language proficiency, social connectedness, gaining educational qualifications, learning how to drive, and securing meaningful employment. Noticeably, many evacuees spoke of the desire to build their capabilities and capacities to become independent and give back to society. Given the challenges evacuees faced when resettling in NZ, they were very grateful for the support provided by NFACT. The success of NFACT’s programme was primarily attributed to the holistic and tailored services provided that addressed evacuees’ resettlement needs, the altruistic nature of staff, and the trusting relationships staff developed with evacuees that was built upon shared lived experience, language, culture, and/or values. NFACT’s comprehensive approach resonates with the broader view of integration and resettlement success employed at the national and international levels [1, 13].

The evacuation of Afghan nationals was unprecedented in some ways and as such, there was limited guidance of how to support evacuees’ resettlement. Other high-income countries, such as Japan and the US, also noted insufficient policies conducive to responding to the humanitarian crisis that was unfolding in Afghanistan [9, 28, 35]. Moreover, Afghan evacuees were not granted official refugee status in NZ; thus, the circumstances within which they were evacuated to NZ may have influenced their experiences and expectations of resettlement services. As such, one of the main challenges that NFACT faced was the speed and scale of the services they had to offer within an uncertain and resource constrictive environment. Despite these resourcing constraints, evacuees found NFACT’s settlement programme to be very helpful with supporting their integration. Evacuees would overwhelmingly recommend the programme to others with migrant, refugee, and refugee-like backgrounds arriving in NZ. Across the datasets, participants’ suggestions for improvement reflected the need for more and longer-term support that matched evacuees’ dynamic, intersecting, and evolving resettlement needs.

### Markers and Means

Evacuees spoke to resettlement challenges they faced, particularly related to educational and employment opportunities, financial pressures, housing, and health, which is similar to Afghan evacuees who resettled in Japan [28]. Similar to the current study, a study undertaken in the United States with Afghan refugees also noted the difficulties with reshaping resettlement expectations particularly given the extended disruptions in education and employment trajectories [35]. Overall, while evacuees were grateful to be safe, their long-term futures were quite uncertain despite being resettled in high-income countries [28, 35].

Interviewed evacuees spoke to the strong desire to build their knowledge and skills. Comprehensive educational services are critical to successful resettlement programmes and long-term outcomes [29]. NFACT supported evacuees by enrolling school-aged children and youth in school. Given the importance of education in Afghan culture, NFACT also ran tailored programmes for youth about university entry requirements and for others to develop new skills. However, like another study, interviewed evacuees spoke to the challenges of learning in an unfamiliar educational environment and how language barriers impeded their learning [30]. For instance, our study shed light on how lack of formal documentation of refugee status and the placement in ESOL classes instead of mainstream classes hindered students’ education.

Evacuees also expressed the importance of engaging in meaningful work, similar to another study [30]. Literature points to how men from refugee backgrounds face pressure related to gender roles and ability to financially provide for their families [44]. In this study, the desire to be financially stable was important to both men and women. This difference may be due to the educational and employment background of interviewee evacuees as many held influential roles in Afghanistan, which were tied to their sense of identity and purpose. The difference could also be related to how interviewed men and women both viewed meaningful work as a mechanism to feel a sense of belonging. Evacuees’ overall goal was to become financially independent thereby being able to support their family and not having to rely on social support and give back to the wider community and society. The desire to become self-sufficient and conceptualising successful resettlement in broader community terms beyond employment resonates with previous findings [35].

While evacuees reported to be in generally good health in the survey, their narratives reflected more nuance as they described the specific health issues that they and their families faced that required specialised support. In addition to supporting evacuees to engage with local general practitioners and other health services, NFACT also provided counselling and emotional wellbeing support. The need for mental health support was particularly evident as evacuees spoke to chronic stress, depression, and anxiety, which is consistent with other studies [9, 28, 30]. Recent literature has called for national mental health policy and services to be guided by a human-rights perspective and community-based responses to be inclusive and responsive to the needs of refugee background individuals [2, 43].

Across the datasets, it was evident that safe, appropriate, and affordable housing was a central resettlement priority for evacuees. Disappointment about housing has also been expressed by refugees entering NZ through the quota programme [23]. Similar housing challenges were also raised among Afghan evacuees given the long hotel stays before moving into the community, long social housing waitlists, and difficulties in the rental housing market [30]. These narratives speak to the financial pressures and discrimination that Afghan evacuees face when trying to secure appropriate and affordable housing. Housing is needed for basic shelter in addition to contributing to stability, social inclusion, and access to services for resettled populations [7, 46]. Evacuees expressed the need for more support with finding suitable housing and information on tenancy rights and responsibilities. With the recognition that housing is an important social determinant of health, NFACT staff supported evacuees with their housing needs despite this not being a part of their contractual obligations.

### Social Connection

Evacuees in our study spoke of the difficulties they experienced when adjusting to life in NZ as this was far from the more affluent and vibrant lives they were accustomed to in Afghanistan. It was evident that developing social connections, particularly across ethnic and cultural groups, was difficult for evacuees and this contributed to feelings of loneliness and isolation. Our study found that developing cross-cultural relationships were especially difficult for those that were older and those with less English proficiency. While Japan’s culturally homogenous society contributed to evacuees feeling marginalised [28], this was also evident in our study despite NZ being considered an ethnically diverse country [39–41]. NFACT’s events that brought people together (e.g., events with locals, cultural and religious celebrations) were invaluable for evacuees to foster connections within and across cultural and ethnic groups. Moreover, participants noted how NFACT’s programmes supported evacuees to become more familiar with the local culture, Māori worldviews, services available, and their rights.

### Facilitators

Similar to previous studies, evacuees in our study experienced challenges due to language barriers and cultural differences [28, 35]. Culturally-appropriate resettlement programmes are pivotal to support integration [28, 35]. The socio-ethnic-cultural-linguistic landscape in Afghanistan represents much diversity, so having staff with lived experience who either shared or were familiar with evacuees’ language and culture was integral to the success of NFACT’s resettlement programme. As access to services was a key barrier among evacuees [30], NFACT developed a comprehensive orientation programme so evacuees could learn about health services, NZ education system, work and income support, housing/tenancy obligations and rights, and how to access emergency and police services. NFACT intentionally developed additional programmes that catered to evacuees’ age and gender specific needs, such as football sessions for youth and sewing classes for women. For families, research has pointed to challenges Afghan refugees face in relation to isolation and lack of multigenerational family support when parenting in a new physical and cultural environment [34]. NFACT offered positive parenting courses to support evacuees, which were well received. Moreover, as gender roles are different in Afghanistan and NZ, a few evacuees recommended sessions that focused on women’s rights which is similar to previous research [35].

### Foundation

Similar to previous research, evacuees in our study experienced difficulties with adjusting to new government and social services and systems [28]. Previous research with quota refugees has recommended having an organisation available for refugees to access should they experience concerns with service providers [23]. NFACT was such an organisation that offered additional programmes that were helpful for evacuees to navigate public services to access support. Moreover, NFACT was viewed as a credible organisation and staff leveraged their connections with key agencies to ensure the needs of evacuees were addressed. Evacuees viewed having NFACT act as an intermediary was crucial to support their resettlement, particularly given the language barriers they faced. Similar to our study, the challenge of not having formal refugee status and the ambiguity of service eligibility on the visa category within which Afghan evacuees entered NZ on led to delays in evacuees being able to access services from government and social service agencies [30].

All survey participants expressed a desire to apply for citizenship and learn more about their rights in NZ. It is important that NZ offered Afghan evacuees a pathway to residency (and eventually citizenship) [12], as this differed from the United States where Afghan arrivals had no pathway to permanent residency [35]. Pathways for residency and citizenship for evacuees are vital to support belonging and create a successful future in their new home country [35]. Moreover, evacuees were separated from their immediate family members, in some cases their children, which contributed to their mental health issues including depression, stress, and anxiety as they feared for their safety and desperately missed the family connections [30]. Although not being able to bring all their children follows Immigration New Zealand’s criteria related to dependent children, it was very difficult for the parents and siblings who were separated from their loved ones. As such, evacuees were eager for more immigration information about and support for family reunification pathways [30, 35].

## Recommendations

Overall, this study provides novel insights into the experiences of Afghan evacuees in accessing resettlement support in NZ. We outline three recommendations to enhance integration and improve resettlement outcomes for individuals with refugee-like backgrounds who are resettling for humanitarian reasons (Table 6).

**Table 6:** Recommendations for improving humanitarian evacuation response and resettlement programmes

Improve readiness to respond to humanitarian crises	• NZ Government should take a more proactive approach in preparing to receive and integrate individuals and families with refugee-like backgrounds in the context of humanitarian evacuations • This should include pathways for permanent residency and citizenship, along with immigration support for family reunification • These plans should be developed in genuine collaboration with communities that have lived experience and intersecting social identities • These plans should leverage multi-sectoral agency collaboration to enhance the quality and efficiency of the support provided
Extend and expand resettlement programmes	• Resettlement support should be provided for a longer duration, with flexibility to address evolving needs over time. Previous research has also called for extended support, recommending a minimum of five years, as assistance during this period can lead to significant long-term improvements [18] • Resettlement support should be holistic and tailored to the changing needs throughout one’s resettlement journey to achieve better future outcomes
Invest in community-led organisations and professional workforce with lived experience	• The NZ Government should invest in community-led organisations to deliver resettlement programmes to enable the delivery of culturally-appropriate resettlement programmes • These organisations can leverage their connections, particularly with key health and social services, to ensure the needs of evacuees were addressed. Previous research has also highlighted the importance of interdisciplinary collaboration across agencies and levels of support in the context of supporting Afghan evacuees [9] • Community-led organisations can also support relationships between tangata whenua (Indigenous people of the land) and tangata Tiriti (people of Te Tiriti o Waitangi) • The professional workforce (e.g., social workers, counsellors, community navigators, etc.) should reflect the diversity of lived experience and social identities while also being proficient in culturally safe practices

### Strengths and Limitations

This study combined quantitative and qualitative methods within a transformative paradigm to improve support for humanitarian resettlement. Strengths of the study include triangulated insights from programme staff and Afghan evacuees. To support efforts to drive change, findings have been shared via an executive summary [4], short technical report [5], and presentations to community, government, and academic audiences.

We recognise that relying on a convenience sample from a small population sub-group may introduce sampling bias and limit generalisability. This pragmatic decision was warranted given the accessibility constraints and project funding timelines. We also acknowledge that our recruitment approach may have introduced selection bias. While our diverse sample of Afghan evacuees reflected variation in age, gender, ethnicity, language, and education, the findings from our participants may not reflect the views of all resettled Afghan evacuees. For the survey, more participants identified as female and Hazara. However, post-stratification for gender found trivial differences in most areas, except for understanding local ways and culture. Moreover, those that chose to participate may have experienced difficulties with offering critical or negative feedback thereby potentially introducing response bias favouring positive commentary. To help mitigate this, no NFACT staff were involved in recruitment and data collection, and participants were explicitly reassured of their confidentiality and encouraged to share their thoughts. Lastly, we note that it is difficult to attribute success of NFACT’s programme in our study as we did not include a comparison group.

### Future Research

Future research should explore evacuees’ migration journeys and resettlement experiences, as well as conduct longitudinal studies to examine long-term integration outcomes for Afghan evacuees. With the understanding that integration as a multi-directional process, research with local communities is also recommended to understand their role in supporting resettlement [14]. Lastly, our study used a widely adopted refugee integration framework [1] that was recently extended to include additional domains like leisure and communication and digital skills [24]. However, agencies currently develop their own success indicators for these domains, highlighting a need for further research. To ensure these indicators reflect lived experiences, these research efforts should be led by and involve those with diverse migration and displacement backgrounds. Moreover, future research could focus on evacuees as a distinct group and develop a tailored conceptual framework to guide integration in the context of humanitarian evacuations.

## Conclusion

This mixed-methods evaluation highlights the success of NFACT’s resettlement programme in supporting Afghan evacuee’s integration. Evacuees expressed a high level of satisfaction with the programme in addressing their resettlement needs, emphasising the importance of shared values and multi-sector collaboration. To achieve better outcomes for those being resettled for humanitarian reasons, we recommend the NZ Government develop clear guidelines, expand programmes, and invest in community-led organisations and a skilled workforce with lived experience.

## Data Availability

The datasets generated and analysed in relation to the presented study are not publicly available due to privacy and ethical reasons. The collected data is of a sensitive and personal nature, and was collected from participants on the basis that strict confidentiality would be maintained. Data can be available from the corresponding author on reasonable request and will require completion of relevant confidentiality agreements.

## References

[1] Ager A, Strang A. Understanding integration: a conceptual framework. J Refug Stud. 2008;21(2):166–91. 10.1093/jrs/fen016.

[2] Brannelly T, Bhatia A, Malihi AZ, Vanderpyl L, Brennan B, Gonzalez Perez L, Saeid F, Holroyd E, Charania N. Refugees and mental wellbeing. A call for community approaches in Aotearoa New Zealand. Ment Health Soc Inclus. 2024. 10.1108/mhsi-04-2024-0049.

[3] Braun V, Clarke V. Successful qualitative research: a practical guide for beginners. London: SAGE; 2013.

[4] Charania NA, Zeng I, Kumar P, Gaylor C, Holroyd E. A mixed-methods evaluation of the Afghan evacuee resettlement programme in Aotearoa New Zealand [executive summary]*.* Migrant and Refugee Health Research Centre, Auckland University of Technology, Auckland. 2024a.

[5] Charania NA, Zeng I, Kumar P, Gaylor C, Holroyd E. A mixed-methods evaluation of the Afghan evacuee resettlement programme in Aotearoa New Zealand [report]*.* Migrant and Refugee Health Research Centre, Auckland University of Technology, Auckland. 2024b 10.1007/s10903-025-01752-4 PMC12882939 40892360

[6] Creswell JW, Plano Clark VL. Designing and conducting mixed methods research. 3rd ed. SAGE; 2017.

[7] Farmer Y. Factors and ethical values that foster a sense of belonging toward the host society: the case of south Asian communities in Montreal’s Parc-Extension neighbourhood (Canada). New Diversities. 2021;23(1):89–103.

[8] Folkes L. Moving beyond ‘shopping list’ positionality: Using kitchen table reflexivity and in/visible tools to develop reflexive qualitative research. Qual Res. 2022;23(5):1301–18. 10.1177/14687941221098922.

[9] Frumholtz M, Carlson WC, Shannon PJ, Iaquinta S, Eckerstorfer M, Hendel-Paterson B, Quadri N, Shetty R, Mohammadzadah H, Stauffer W, Adesida O, Howard C, Urban K, Kirsch J, Sayad M, Mamo B. Welcoming new neighbors: Minnesota’s rapid response model to address the urgent health needs of Afghan newcomers, 2021–2022. Front Public Health. 2024;12: 1413258. 10.3389/fpubh.2024.1413258. 38989114 10.3389/fpubh.2024.1413258 PMC11233686

[10] Harrison RL, Reilly TM, Creswell JW. Methodological rigor in mixed methods: an application in management studies. J Mixed Methods Res. 2020;14(4):473–95. 10.1177/1558689819900585.

[11] Hsieh HF, Shannon SE. Three approaches to qualitative content analysis. Qual Health Res. 2005;15(9):1277–88. 10.1177/1049732305276687. 16204405 10.1177/1049732305276687

[12] Immigration New Zealand. Residence pathway for Afghan evacuees. 2023. https://www.immigration.govt.nz/new-zealand-visas/preparing-a-visa-application/living-in-new-zealand-permanently/afghan-national-resettlement/residence-pathway-for-afghan-evacuees. Accessed 21 Feb 2023.

[13] Immigration New Zealand. New Zealand refugee resettlement strategy. 2024. https://www.immigration.govt.nz/about-us/what-we-do/our-strategies-and-projects/refugee-resettlement-strategy. Accessed 19 Sept 2024.

[14] Klarenbeek LM. Reconceptualising ‘integration as a two-way process.’ Migr Stud. 2021;9(3):902–21. 10.1093/migration/mnz033.

[15] Lincoln Y, Guba EG. Naturalistic inquiry. Newbury Park: SAGE; 1985.

[16] Manatū Hauora - Ministry of Health. Te Pae Mahutonga model of Māori health. 2017. https://www.health.govt.nz/maori-health/maori-health-models/te-pae-mahutonga.

[17] Manatū Hauora - Ministry of Health. Te Whare Tapa Whā model of Māori health. 2023. https://www.health.govt.nz/maori-health/maori-health-models/te-whare-tapa-wha.

[18] Marlowe J, Malihi AZ, Milne B, McLay J, Chiang A. Settlement trajectories of nearly 25,000 forced migrants in New Zealand: longitudinal insights from administrative data. Kotuitui New Zeal J Soc Sci Online. 2023;19(1):21–44. 10.1080/1177083x.2023.2214606.

[19] Mastaglia B, Toye C, Kristjanson LJ. Ensuring content validity in instrument development: challenges and innovative approaches. Contemp Nurse. 2003;14(3):281–91. 10.5172/conu.14.3.281. 12868667 10.5172/conu.14.3.281

[20] McAdam J. Evacuations: a form of disaster displacement? Forced Migr Rev. 2022;69:56–7. 10.1093/rsq/hdaa017.

[21] Mertens DM. Research and evaluation in education and psychology: Integrating diversity with quantitative, qualitative, and mixed methods. 6th ed. London: SAGE Publications Inc.; 2024.

[22] Mertens DM, Bledsoe KL, Sullivan M, Wilson A. Utilization of mixed methods for transformative purposes. In: Tashakkori A, Teddlie C, editors. SAGE handbook of mixed methods in social and behavioral research. SAGE Publications, Inc.; 2010.

[23] Nakhid C, Moulvi N. An evaluation of the settlement services provided to former refugees arriving via the Refugee Quota Programme. Migrant Action Trust - Ethnic Communities Advocacy and Research Unit. 2023.

[24] Ndofor-Tah C, Strang A, Phillimore J, Morrice L, Michael L, Wood P, Simmons J. Home Office indicators of integration framework 2019. Home Office (London); 2019.

[25] New Settlers Family and Community Trust. Annual Report 2022–23. 2023a. https://www.nfact.co.nz/blog/post/111507/annual-report/. Accessed 21 Feb 2023.

[26] New Settlers Family and Community Trust. New Settlers Family and Community Trust (NFACT). 2023b. https://www.nfact.co.nz/. Accessed 21 Feb 2023.

[27] Nguyen TD, Attkisson CC, Stegner BL. Assessment of patient satisfaction: development and refinement of a service evaluation questionnaire. Eval Program Plann. 1983;6:299–314. 10267258 10.1016/0149-7189(83)90010-1

[28] Ogawa R, Ahmad ZH, Hourieh A. The experience of becoming a refugee: evacuation and resettlement of Afghanistan citizens in Japan. THINK Lobby J. 2024;2:89–101.

[29] Okoye, Cynthia. A Scan of Settlement Programs and Services in Western Canada: Integration of Newcomers in Prince George, British Columbia; 2020.

[30] Papoutsaki E, Bhana A. Aotearoa New Zealand government evacuation and resettlement support for the 2021 Afghanistan crisis: Project findings*.* World Vision, ActionStation, and Amnesty International Aotearoa New Zealand. 2023. https://www.worldvision.org.nz/getmedia/9f9bd2f6-e8bc-45d3-88dd-b59361c83a72/WV-Afghanistan-Research-Report-Final-Digital_1.pdf

[31] Puma JE, Lichtenstein G, Stein P. The RISE survey: developing and implementing a valid and reliable quantitative measure of refugee integration in the United States. J Refug Stud. 2018;31(4):605–25. 10.1093/jrs/fex047.

[32] Red Cross New Zealand. First anniversary of Afghan evacuees arriving in New Zealand. 2022. https://www.redcross.org.nz/about-us/news/our-stories/first-anniversary-of-afghan-evacuees-arriving-in-new-zealand/. Accessed 21 February 2023.

[33] Reeve C, Humphreys J, Wakerman J. A comprehensive health service evaluation and monitoring framework. Evaluation Program Planning. 2015;53:91–8. 10.1016/j.evalprogplan.2015.08.006. 26343490 10.1016/j.evalprogplan.2015.08.006

[34] Rosenberg J, Leung JK, Harris K, Abdullah A, Rohbar A, Brown C, Rosenthal MS. Recently-arrived Afghan refugee parents’ perspectives about parenting, education and pediatric medical and mental health care services. J Immigr Minor Health. 2022;24(2):481–8. 10.1007/s10903-021-01206-7. 33934263 10.1007/s10903-021-01206-7 PMC12335978

[35] Salley E. Reaching actualisation after resettlement: A qualitative analysis of Afghan refugees in the U.S. Peace Human Rights Governance. 2024;8(1):103–132. 10.14658/PUPJ-PHRG-2024-1-5.

[36] Sandelowski M. Sample size in qualitative research. Res Nurs Health 1995;18(2):179–83. 10.1002/nur.4770180211. https://www.ncbi.nlm.nih.gov/pubmed/7899572. 10.1002/nur.4770180211 7899572

[37] Sandelowski. Whatever happened to qualitative description? Res Nurs Health 2000;23(4):334–40. https://www.ncbi.nlm.nih.gov/pubmed/10940958. 10.1002/1098-240x(200008)23:4<334::aid-nur9>3.0.co;2-g 10940958

[38] Sandelowski. What’s in a name? Qualitative description revisited. Res Nurs Health 2010;33:77–84. 10.1002/nur.20362 20014004

[39] Statistics NZ. First results from the 2023 Census – older, more diverse population, and an extra 300,000 people between censuses. 2024a. https://www.stats.govt.nz/news/first-results-from-the-2023-census-older-more-diverse-population-and-an-extra-300000-people-between-censuses/. Accessed 24 Sept.

[40] Statistics NZ. Net migration remains near record level. 2024b. https://www.stats.govt.nz/news/net-migration-remains-near-record-level/.

[41] Statstics NZ. Population projected to become more ethnically diverse. 2021. https://www.stats.govt.nz/news/population-projected-to-become-more-ethnically-diverse/. Accessed 24 Sept.

[42] United Nations High Commissioner for Refugees. Handbook for the protection of internally displaced persons, Guidance Note 9: Humanitarian Evacuations*.* 2010. https://www.unhcr.org/media/handbook-protection-internally-displaced-persons-0.

[43] Van derpyl L, Charania N, Treharne GJ, Al Naasan Z. The potential of a rights-based approach to refugee-focused mental health policy in Aotearoa New Zealand. Kōtuitui New Zeal J Soc Sci Online. 2024. 10.1080/1177083X.2024.2404057.

[44] West-McGruer K, Karabadogomba JP, Chaol G, Pau J, Sivanathan S, Yor A, Humpage L, Marlowe J. Improving resettlement outcomes: What can we learn from men from refugee backgrounds?*.* Auckland Resettled Community Coalition, Auckland; 2018.

[45] Williams KE, McMahon T, Grech K, Samsa P. Resettlement factors associated with subjective well-being among refugees in Australia: findings from a service evaluation. J Immigr Refuge Stud. 2024;22(1):52–67. 10.1080/15562948.2021.1996671.

[46] Ziersch A, Miller E, Baak M, Mwanri L. Integration and social determinants of health and wellbeing for people from refugee backgrounds resettled in a rural town in South Australia: a qualitative study. BMC Public Health. 2020;20:1–16. 33187489 10.1186/s12889-020-09724-z PMC7663864

